# EEG Baseline Analysis for Effective Extraction of P300 Event-Related Potentials for Welfare Interfaces

**DOI:** 10.3390/s25103102

**Published:** 2025-05-14

**Authors:** Yuta Sasatake, Kojiro Matsushita

**Affiliations:** 1 Intelligent Production Technology Research & Development Center for Aerospace, Institute for Advanced Study, Gifu University, Gifu 501-1193, Japan; 2 Department of Mechanical Engineering, Gifu University, Gifu 501-1193, Japan; matsushita.kojiro.h7@f.gifu-u.ac.jp

**Keywords:** EEG, BCI, ERP-P300, baseline

## Abstract

Enabling individuals with complete paralysis to operate devices voluntarily requires an effective interface; EEG-based P300 event-related potential (ERP) interfaces are considered a promising approach. P300 is an EEG peak generated in response to specific sensory stimuli recognized by an individual. Accurate detection of this peak necessitates a stable pre-stimulus baseline EEG signal, which serves as the reference for baseline correction. Previous studies have commonly employed either a single-time-point amplitude (e.g., at 100 ms before stimulus onset) or a time-range-averaged amplitude over a specified pre-stimulus period (e.g., 0–200 ms) as a baseline correction method, assuming these provide the most stable EEG reference. However, in assistive P300 interfaces, continuous visual stimuli at 400 ms intervals are typically used to efficiently evoke P300 peaks. Since stimuli are presented before the EEG stabilizes, it remains unclear whether conventional neuroscience baseline correction methods are suitable for such applications. To address this, the present study conducted a P300 induction experiment based on continuous 400 ms interval visual stimuli. Using EEG data recorded from 0 to 1000 ms before each visual stimulus (sampled at 1 ms intervals), we applied three baseline correction methods—single-time-point amplitude, time-range-averaged amplitude, and multi-time-point amplitude—to determine the most effective EEG reference and evaluate the impact on P300 detection performance. The results showed that baseline correction using an amplitude at a single point in time is unstable when the basic EEG rhythm and low-frequency noise remain, while time-range-averaged baseline correction using the 0–200 ms pre-stimulus period led to relatively effective P300 detection. However, it was also found that using only one value averaged over the amplitude from 0 to 200 ms did not result in an accurate EEG reference potential, resulting in an error. Finally, this study confirmed that the multi-time-point baseline correction method, through which the amplitude state from 0 to 200 ms before the visual stimulus is comprehensively evaluated, may be the most effective method for P300 determination.

## 1. Introduction

In the fields of healthcare and welfare, human–machine interfaces (HMIs) that utilize bio-signals, such as EEG, electromyography (EMG), and electrooculography (EOG), are essential [1,2]. Among these, interfaces based on EEG signals are known as brain–computer interfaces (BCIs), which serve as the sole means for individuals with severe conditions such as amyotrophic lateral sclerosis (ALS), brainstem infarction, cerebral palsy, and spinal cord injuries to control a computer [3,4]. BCIs can be categorized as invasive or non-invasive based on the method of EEG measurement. Examples of invasive BCIs include electrocorticography (ECoG), which involves placing electrodes on the brain surface, and local field potentials (LFPs), which require electrodes to be implanted directly in the brain [5]. These methods necessitate surgery to open the skull and dura mater for electrode placement, imposing a significant burden on the user. However, they allow for the direct measurement of brain signals, yielding high temporal and spatial resolutions, which enables real-time multi-input control [6,7,8]. In contrast, non-invasive BCIs commonly utilize scalp EEG [9], in which electrodes are placed on the scalp [10,11,12]. This approach does not require surgery, making it more accessible for daily use, though some of the signal strength is attenuated as it passes through the skull and scalp. Consequently, a single EEG recording is insufficient to achieve a reliable control input. Instead, non-invasive BCIs typically analyze 40–120 EEG epochs, resulting in one input command approximately every 30–60 s, which limits real-time control. Although the performance of non-invasive BCIs is generally lower than that of invasive methods, the ease of use and the non-surgical nature make scalp EEG-based BCIs promising for practical applications [13].

Scalp EEG-based BCIs can be divided into two categories: those that detect periodic bio-signals, known as “baseline rhythms”, and those that capture event-related potentials (ERPs), which are transient signal peaks generated when the brain recognizes specific sensory stimuli [14]. Among ERPs, P300 is the only waveform with successful applications in practical scalp EEG-based BCI systems. P300 is an EEG peak that appears as a positive deflection approximately 300 ms after a person attends to a particular sensory stimulus presented randomly and at a fixed frequency [15,16]. Leveraging this characteristic, Farwell and Donchin developed a P300-based speller interface in which 26 letters flash alternately on a screen. By analyzing the timing of the letter flashes and ERP responses, they were able to infer the letter the user focused on, creating a keyboard input BCI known as the P300 speller [17]. Many studies have further developed the P300 speller. One direction of research has aimed to improve sensory presentation methods to enhance users’ recognition of the target stimulus. For example, Kirasirova and colleagues found that flashes of letters surrounding the target letter could negatively affect the P300 peak for the target. By limiting the visual field, they improved the detection accuracy [18]. Similarly, Kaufmann et al. achieved higher recognition accuracy by replacing letter flashes with images of human faces, which are more readily recognizable by users [19]. Another line of research has focused on refining methods for extracting the P300 peak. The foundation for P300 analysis, proposed by Galambos and Sheatz in 1964 [20], involves the following process: first, EEG data are segmented into epochs from 1 s before to 1 s after stimulus onset, and these epochs are categorized by stimulus type. Next, baseline correction is applied, setting the most stable EEG point within each epoch as the zero-voltage reference (EEG baseline). Typically, either the amplitude at a specific pre-stimulus time (e.g., 100 ms; single-time-point baseline correction) or the average amplitude over a pre-stimulus period (e.g., 0–200 ms; time-range-averaged baseline correction) is used as the zero point, with all the EEG data adjusted accordingly. Finally, the averaged waveform for each stimulus type is calculated. This process allows for the cancellation of baseline rhythms (around 50 µV) that obscure small P300 peaks (around 5 µV) by taking advantage of the phase differences in baseline rhythms across epochs [21,22,23].

Thus, P300 detection accuracy improves with longer experiment durations and more epochs for averaging. However, in scenarios involving continuous sensory presentation, such as with the P300 speller at 200 ms intervals, achieving a stable baseline reference may be challenging, potentially reducing detection accuracy. To address this, Tanner and Norton proposed improving baseline correction by simultaneously recording EEG and magnetoencephalography (MEG) during ERP-evoking stimuli. They clarified the effective high-pass filter settings for baseline correction, assuming MEG as the accurate reference [24].

Additionally, Krusienski et al. explored using additional EEG sites (PO7, PO8, and Oz) beyond the standard P300 sites (Fz, Cz, and Pz), enhancing the baseline reference detection across multiple channels [25]. Furthermore, in recent years, several studies have been conducted on P300 detection using machine learning based on convolutional neural networks (CNNs) as an advancement of the P300 speller. Kilani et al. demonstrated that by training a model directly on the EEG waveforms of target and non-target stimuli without applying conventional baseline correction or averaging, it is possible to detect the presence or absence of the P300 component in raw EEG signals [26]. However, in order to achieve a high classification accuracy, subject-specific training (fine-tuning) is necessary due to inter-individual differences in EEG waveform characteristics. In contrast, Li et al. proposed a machine learning-based P300 detection method that does not require fine-tuning [27]. Their approach involved training on EEG waveforms after applying time-range averaged baseline correction and averaging, enabling a high classification performance without subject-specific training. This indicates that baseline correction and averaging effectively reduce individual variability in EEG waveforms, highlighting the critical role of these preprocessing techniques in P300 detection.

Given the continuous 400 ms interval sensory stimuli used in P300-based BCIs, the characteristics of the EEG baseline potential under such conditions have not been thoroughly analyzed. Additionally, the effectiveness of conventional baseline correction methods, such as single-time-point baseline correction and time-range-averaged baseline correction, has not been quantitatively validated. Therefore, in this study, we conducted a P300 evoked potential experiment with continuous 400 ms visual stimuli, measuring EEG data from 0 ms to 1000 ms before stimulus onset (with a 1 ms resolution). Three types of baseline correction (single-time-point, time-range-averaged, and multi-time-point) are applied to analyze P300 peaks. We then analyzed a baseline method for calculating EEG reference potentials that would allow effective P300 detection for continuous stimulation.

In Experiment 1, we performed single-time-point baseline correction for all time points between 0 ms and 1000 ms before visual stimuli, analyzed all waveforms, and examined the characteristics of the single-time-point baseline method that can effectively determine P300. In Experiment 2, we applied time-range-averaged baseline correction using different baseline durations ranging from 0 ms to 1000 ms before stimulus onset, analyzed all the waveforms, and examined the characteristics of the time-range-averaged baseline correction that can effectively determine P300. In Experiment 3, we test our proposed multi-time-point baseline correction to provisionally determine the P300 for all points in a specific time range using single-time-point baseline correction, and then evaluate the results of these judgments comprehensively and make a final judgment. We then analyze all waveforms and verify that the multi-time-point baseline correction can effectively determine P300.

## 2. Materials and Methods

### 2.1. Overview of the EEG Measurement Experiment

**Figure 1:**
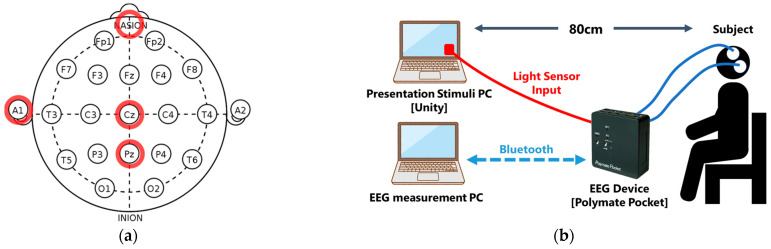
Electrode placement and overview of the experimental setup. (**a**) Electrode placement based on the international 10–20 system, showing the ground electrode on the forehead, the reference electrode on A1 (left earlobe), and the recording electrodes at Cz and Pz. Red circles indicate the electrodes used in this study; (**b**) overview of the experimental apparatus.

Participants were seated on a chair positioned 80 cm away from the PC used for visual stimulus presentation. EEG signals were recorded using the Polymate Pocket device (Miyuki Giken Co., Ltd., Tokyo, Japan), with electrodes placed according to the international 10–20 system: a ground electrode on the forehead, a reference electrode on A1 (left earlobe), and recording electrodes at Cz and Pz. All electrode–skin impedances were maintained below 30 kΩ (Figure 1a). Additionally, to synchronize the visual stimulus onset with EEG recordings, the voltage output of a photosensor attached to the display was connected to the external input pins of the EEG device. This setup allowed the detection of visual stimulus timing based on changes in the color of a specified area on the display (Figure 1b).

During the experiment, participants were instructed to minimize head movements and to focus on a single designated visual stimulus per trial. The EEG signals measured from the electrodes first passed through a 0.03 Hz high-pass filter integrated into the EEG device circuitry. Subsequently, the signals were filtered by an anti-aliasing low-pass filter with a cutoff frequency of 333 Hz and were recorded onto a PC at a sampling rate of 1 kHz. Additionally, a second-order Butterworth high-pass filter with a cutoff frequency of 0.05 Hz was applied via software to prepare the EEG data for further analysis. Regarding the EEG measurement environment, because this study targets welfare applications, measurements were conducted in a standard room mimicking a typical home or office environment rather than an electromagnetic shielded room. However, the placement of electrical devices powered by commercial electricity was carefully adjusted to prevent the generation of commercial power line noise.

### 2.2. Visual Stimulus Presentation Method

**Figure 2:**
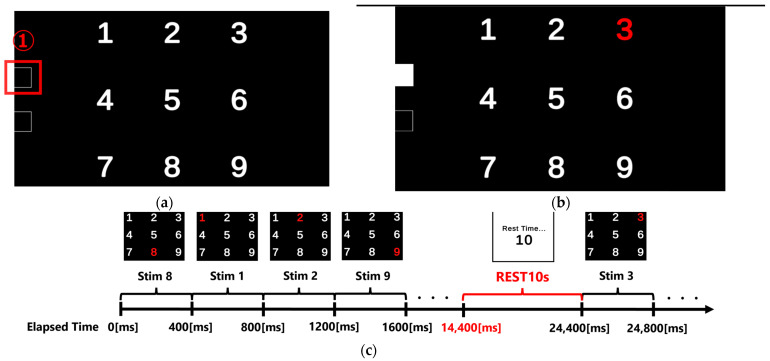
Visual stimuli and time chart of the stimulus presentation. (**a**) Layout of digits presented in a 3 × 3 grid on screen. White digits from 1 to 9 were used as stimuli. The red square (area ①) indicates the location where the photosensor was placed to detect the timing of stimulus onset; (**b**) Example screen displaying the visual stimulus “3”, which changes color from white to red for 200 ms during stimulus presentation; (**c**) graph showing the order of visual stimulus presentations and the flow of the 10-second rest period. Red text and brackets indicate the rest duration (REST10s) and its position on the time axis (e.g., 14,400 [ms]).

Visual stimuli were presented using a Unity program running on a PC with a built-in GPU, displayed on a 27-inch monitor (resolution: 3840 × 2160). The stimulus consisted of white digits from 1 to 9 (stimulus numbers) arranged in a 3 × 3 grid against a black background (Figure 2a). During stimulus presentation, the color of the digit changed from white to red for 200 ms (Figure 2b). Digits from 1 to 9 flashed randomly at 400 ms intervals, and the same digit was not repeated consecutively. The experiment was designed so that, by the end, each digit flashed an equal number of times. To help participants maintain concentration and minimize blinking and body movement, a 10-s break was provided after every 36 stimulus presentations (Figure 2c). Area ① in Figure 2a indicates the location where the photosensor was placed.

### 2.3. P300 Peak Analysis Method

**Figure 3:**
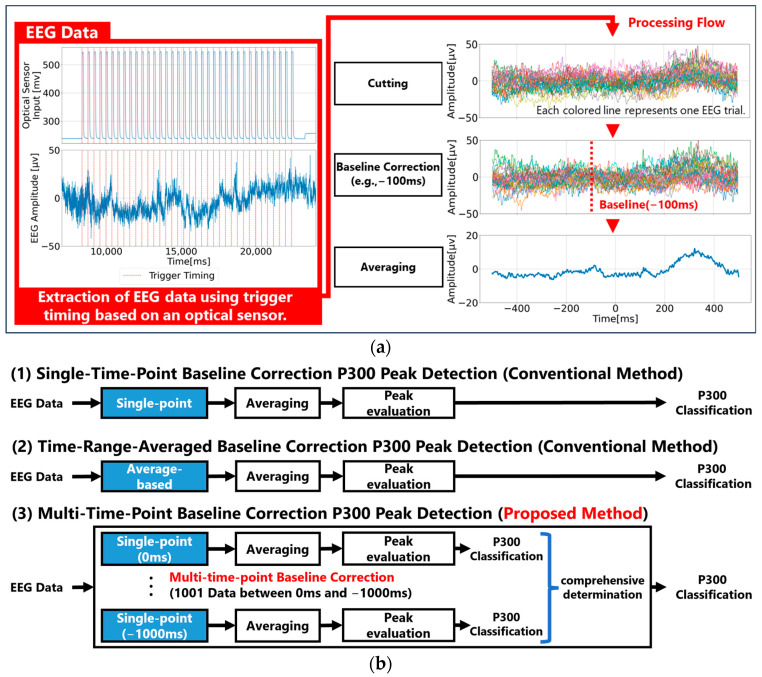
Comparison of P300 peak calculation and detection processes based on baseline correction methods. (**a**) The process from measurement data to P300 peak calculation; (**b**) detection processes for P300 based on conventional single time point and time-range-averaged baseline correction methods compared to the proposed multi-time-point baseline correction method.

P300 peak analysis of EEG and event-related potentials (ERPs) was performed using a Python-based program. As shown in Figure 3a, EEG data were first segmented from 1000 ms before to 500 ms after visual stimulus onset using the change in voltage detected by the photosensor as the stimulus timing reference. After segmenting the EEG data, baseline correction was applied, and averaged ERP waveforms were computed for each type of visual stimulus to confirm the presence of a P300 peak. This study focuses on baseline correction, and the following three baseline correction methods were applied:

1. Single-Time-Point Baseline Correction (Conventional Method)

This method treats a specific time point before stimulus onset (e.g., −100 ms) as the EEG baseline reference voltage. The entire segmented EEG waveform is adjusted so that the amplitude at this single time point becomes 0 V (Figure 3(b1)).

2. Time-Range-Averaged Baseline Correction (Conventional Method)

In this method, the average amplitude over a specific time range before the stimulus (e.g., −200 ms to 0 ms) is treated as the EEG baseline reference voltage. The entire segmented EEG waveform is adjusted so that this average value becomes 0 V (Figure 3(b2)).

3. Multi-Time-Point Baseline Correction (Proposed Method)

In this method, the amplitudes at multiple time points before the stimulus are each treated as EEG baseline reference voltages. Baseline correction is performed for each reference point, and preliminary P300 peak detection is conducted accordingly. Then, all preliminary detection results are aggregated, and the visual stimulus with the highest proportion of P300 peak detections is finally determined as the target stimulus in which the P300 peak is considered to have occurred (Figure 3(b3)). The major difference between this method and conventional methods is that it does not use only a single amplitude value, such as that from a single time point or a time-range-averaged value, as the EEG baseline reference. Therefore, compared to conventional methods that rely on a single value, this method can evaluate the amplitude characteristics of the pre-stimulus EEG data in a more multivariate manner, leading to a more robust estimation of the baseline reference.

The artifact removal process was performed on the data segments extracted based on the visual stimulus timing. To eliminate the effects of electrooculographic (EOG) artifacts and body movements, any extracted data in which the EEG amplitude exceeded the range of ±100 μV was excluded and not included in the subsequent P300 analysis.

### 2.4. Participants and Analyzed Data

In this study, EEG measurements were conducted for 27 healthy participants aged 19 to 37 years (seven females and 20 males). In the first trial, visual stimulus number 3 was set as the target stimulus, and each of the nine visual stimuli was presented 100 times. In the second trial, stimulus number 5 was set as the target, with each stimulus again presented 100 times. Among the 27 participants, data from five participants were excluded from the analysis based on the following criteria:

1. The participant fell asleep during the experiment.
2. The EEG data contained excessive noise due to excessive body movements.
3. There was poor electrode contact, or electrode detachment occurred during the measurement.

As a result, data from the remaining 22 participants (seven females and 15 males, aged 19 to 37 years) across both trials were used for analysis.

## 3. Analysis 1: Characteristics of Conventional Single-Time-Point Baseline Correction for P300 Peak Detection

Single-time-point baseline correction, particularly using the baseline 100 ms before visual stimulus presentation, is the most commonly used method in previous studies for P300 peak detection. However, other studies have employed baselines from 200 ms or even 1000 ms before the stimulus. We extracted EEG data from 1000 ms before to 500 ms after the visual stimulus and applied single-time-point baseline correction at each 1 ms interval from 0 to 1000 ms prior to the stimulus. Baseline-corrected EEG data for each stimulus were then averaged, and the maximum amplitude within a specific post-stimulus time range was extracted. By comparing the maximum amplitudes obtained for each stimulus, we analyzed and discussed the effectiveness of this method for P300 peak detection, aiming to identify the most effective baseline point for amplitude-based baseline correction.

### 3.1. Feature Analysis of the Maximum Amplitude in P300 Peak Detection Based on Single-Time-Point Baseline Correction

**Figure 4:**
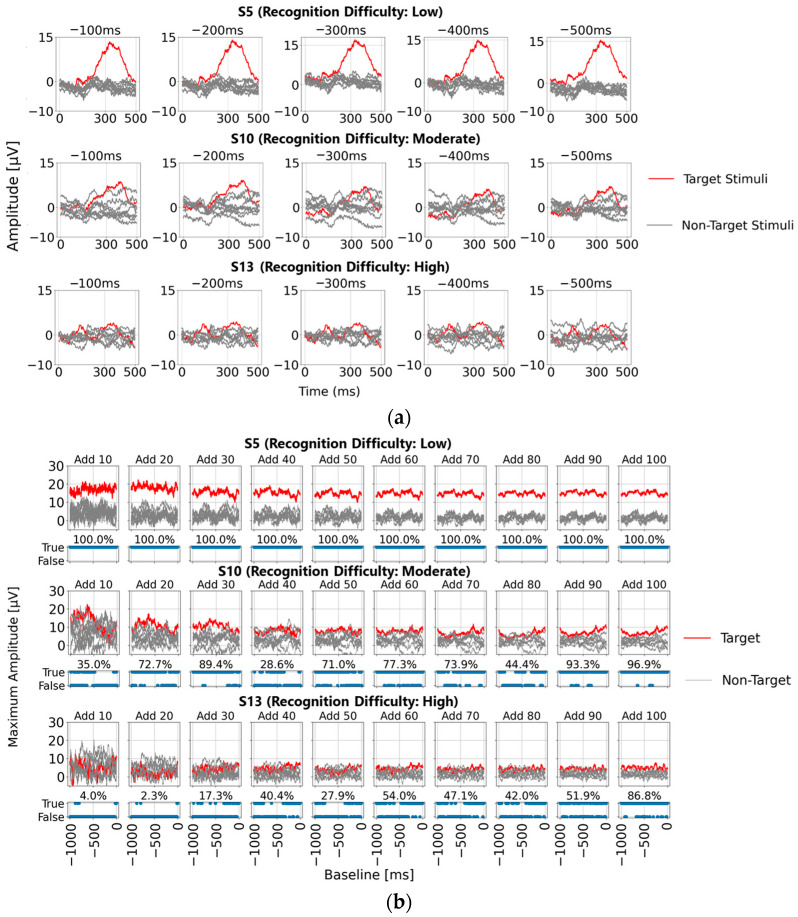

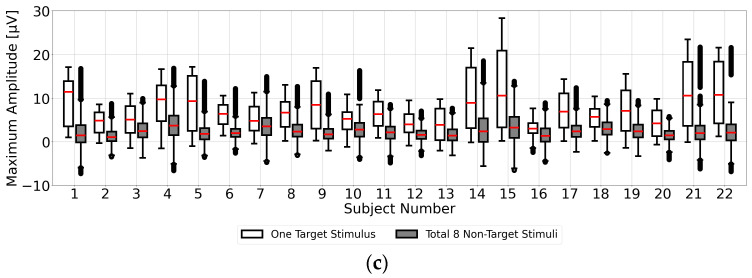
Feature analysis of P300 detection based on single-time-point baseline correction. (**a**) P300 peak waveforms for three participants based on five conventional single-time-point baseline corrections (averaged over 100 trials); (**b**) maximum amplitudes across all baseline points for three participants using single-time-point baseline correction at each time point (top) and the correct/incorrect classifications when the maximum amplitude for the target stimulus exceeded that of the non-target stimulus (bottom), visualized as blue bars labeled “True” for correct and “False” for incorrect classifications and displayed for different averaging trial counts; (**c**) box plot of maximum amplitudes for target and non-target stimuli for all 22 participants. The red horizontal lines within each box indicate the median values. The black markers and whiskers represent outliers and the data range excluding outliers, respectively.

To facilitate comparison, we first focused on five representative baseline points used in single-time-point baseline correction (100, 200, 300, 400, and 500 ms before visual stimulus presentation) and calculated the P300 peak detection results based on each baseline point. Figure 4a shows the post-stimulus EEG waveforms after 100 trials of averaging, classified as “Recognition Difficulty: Low”, “Recognition Difficulty: Moderate”, or “Recognition Difficulty: High”. The waveform of S5 represents a “Recognition Difficulty: Low” outcome, where a clear P300 peak appeared during the target stimulus and the waveform stabilizes near zero during the non-target stimulus. In contrast, for S10 (“Recognition Difficulty: Moderate”) and S13 (“Recognition Difficulty: High”), some baseline points led to higher amplitudes during the non-target stimulus than during the target stimulus, making P300 peak detection challenging. This suggests that conventional single-time-point baseline correction may not provide a stable “EEG baseline voltage” at certain points.

However, this issue could also have arisen due to inadequate suppression of the background rhythm due to insufficient averaging. To investigate further, Figure 4b illustrates the results of applying single-time-point baseline correction at 1 ms intervals from 0 to 1000 ms before the visual stimulus. Maximum amplitudes were calculated within the 200–400 ms post-stimulus range (300 ± 100 ms), with the *x*-axis representing the baseline point and the *y*-axis the maximum amplitude. For each baseline point, correctness was determined if the maximum amplitude during the target stimulus was higher than that during the non-target stimulus. The lower part of each graph shows correct/incorrect classification results, and the accuracy rate is displayed. These results are presented for averaging trials of 10, 20, 30, 40, 50, 60, 70, 80, 90, and 100.

In addition, the top row in Figure 4b illustrates the “Recognition Difficulty: Low” condition, the middle row the “Recognition Difficulty: Moderate” condition, and the bottom row the “Recognition Difficulty: High” condition. Across all conditions, non-target stimulus maximum amplitudes remained high with less than 20 averages, indicating residual background rhythm. Starting from 40 averages, the impact of the background rhythm diminished. In the “Recognition Difficulty: Low” condition of S5, target stimulus P300 peak amplitudes are clearly distinguishable across all baseline points. However, in the “Recognition Difficulty: Moderate” (S10) and “Recognition Difficulty: High” (S13) conditions, non-target stimulus amplitudes remained higher, even with 40 or more averages, and stable P300 peak detection was only achieved after 90 or more averages. Thus, while averaging 40 or more times reduces background rhythm interference, weak P300 peaks from target stimuli can result in incorrect judgments due to unstable EEG baseline voltages at certain baseline points, causing higher maximum amplitudes for non-target stimuli.

Finally, in Figure 4c, for each of the 22 participants, we applied single-time-point baseline correction at 1 ms intervals from 0 to 1000 ms before the visual stimulus. Maximum amplitudes within the 200–400 ms range (300 ± 100 ms) post-stimulus were calculated from the averaged EEG data of 100 trials. Statistical results of the maximum amplitudes for the target (one stimulus) and non-target (eight stimuli) stimuli are shown as box plots. This confirms that the median of the maximum amplitudes from the target stimuli exceeded that from the non-target stimuli across all participants, although higher maximum amplitudes from the non-target stimuli were observed at certain points, potentially leading to misclassification. This suggests the importance of determining a stable EEG baseline voltage point within the range of 0 to 1000 ms prior to the visual stimulus.

### 3.2. Feature Analysis of the Time Range for Determining Maximum Amplitude in the Single-Time-Point Baseline Correction for P300 Peak Detection

**Figure 5:**
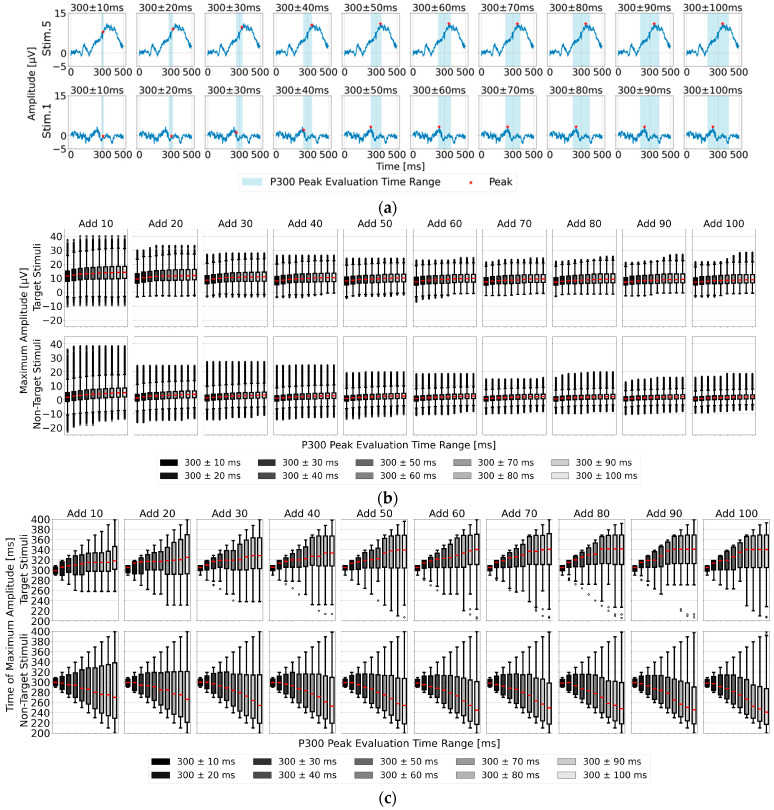
Feature analysis of the time range and maximum amplitude in P300 detection based on single-time-point baseline correction (top: target stimuli; bottom: non-target stimuli). (**a**) Illustration of the time range and maximum amplitude for P300 peak detection in the EEG data of one participant; (**b**) box plot of the maximum amplitudes across the time ranges for P300 peak detection in all 22 participants, displayed for various averaging trial counts; (**c**) box plot of the time positions of the maximum amplitudes within the P300 detection time range for all 22 participants.

As shown in Figure 5a, the position of the P300 peak did not occur exactly at 300 ms after visual stimulus onset but varied between individuals. Additionally, the P300 peak width spans approximately 100 to 200 ms, necessitating an appropriately defined detection time range (P300 peak detection time range) to accurately capture individual differences in the P300 peak position. Therefore, we analyzed EEG data for the 22 participants by applying 10 different time ranges around 300 ms post-stimulus, with intervals of ±10 ms up to ±100 ms. We evaluated both the maximum amplitude used as an indicator for P300 peak detection and the corresponding time position for the target (one stimulus) and non-target stimuli (eight stimuli combined) averaged EEG data. The results are presented as box plots in Figure 5b,c. Regarding the median maximum amplitude in Figure 5a, the values show an upward trend from 300 ± 10 ms to around 300 ± 60 ms, stabilizing in the 300 ± 70 to 100 ms range. In Figure 5b, after averaging 80 trials or more, the time position of the maximum amplitude stabilized particularly within the range of 300 ± 70 to 100 ms, suggesting that 300 ± 70 ms is the most suitable among the 10 time ranges.

Another notable feature is that the maximum amplitude of the target stimulus tended to occur later than 300 ms, while that of the non-target stimulus tended to appear earlier than 300 ms. This characteristic can also serve as a useful indicator for P300 peak detection. Considering these two features—the time range of 300 ± 70 ms and the tendency for the target stimulus maximum amplitude to occur later than 300 ms—the time range of 300 to 370 ms is estimated to be the most effective for detecting the maximum amplitude of the target stimulus.

### 3.3. Detailed Analysis of P300 Peak Detection in the 300–370 ms Time Range for Single-Time-Range Baseline Correction

In the previous section, it was suggested that detecting the P300 peak as the maximum amplitude within the 300–370 ms range after the visual stimulus is the most effective approach. Therefore, in this section, we performed single-time-point baseline correction at each 1 ms interval from 0 to 1000 ms before the visual stimulus for each of the 22 participants. For each baseline-corrected, averaged EEG dataset, the maximum amplitude within the 300–370 ms range post-stimulus was calculated. A detailed statistical analysis was conducted on the maximum amplitudes for the target (one stimulus) and non-target stimuli (eight stimuli) to evaluate the time points that provide a stable EEG baseline voltage within the 0–1000 ms pre-stimulus range.

**Figure 6:**
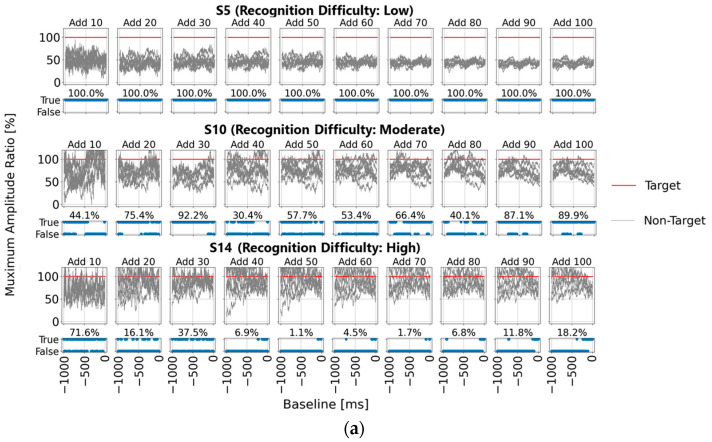

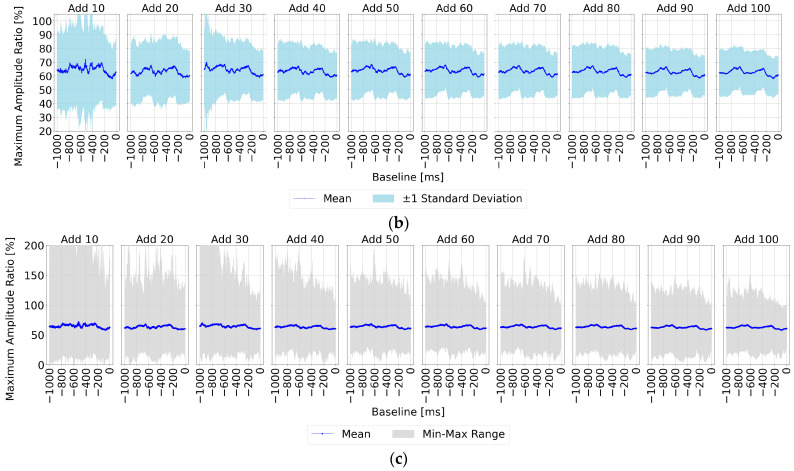
Detailed comparison of the maximum amplitudes for target and non-target stimuli with the P300 detection time range set to 300–370 ms (single-time-point baseline correction). (**a**) Normalized maximum amplitude values for non-target stimuli relative to the target stimulus maximum amplitude, shown for three participants across different averaging trial counts. The correct/incorrect classifications based on these values are visualized as blue bars labeled “True” for correct and “False” for incorrect classifications; (**b**) statistical representation (mean and standard deviation) of the normalized maximum amplitude values for non-target stimuli relative to target stimuli for all 22 participants; (**c**) statistical representation (mean, maximum, and minimum) of the normalized maximum amplitude values for non-target stimuli relative to target stimuli for all 22 participants.

First, Figure 6a shows the ratio of the maximum amplitude for the non-target stimuli to that for the target stimuli (set to 100%) for each baseline time point (horizontal axis), allowing for easy observation of non-target stimulus influence. The upper section of the figure displays the results for averaging trial counts of 10, 20, 30, 40, 50, 60, 70, 80, 90, and 100, while the lower section shows correct/incorrect classifications at each baseline time point along with accuracy rates. Additionally, the results for the three representative participants—S5, S10, and S13—are presented, as in Section 3.1. The findings align with the trends noted in Section 3.1; however, a key observation is that with 90 or more averaging trials, all participants showed lower maximum amplitude ratios for the non-target stimuli compared to the target stimuli within the 0–200 ms range pre-stimulus, indicating a stable EEG baseline voltage.

Subsequently, Figure 6b,c present statistical results across all 22 participants, showing the ratio of non-target to target maximum amplitude values when the target amplitude was set to 100%. Figure 6b displays the mean and standard deviation, while Figure 6c illustrates the mean, maximum, and minimum values within shaded areas. Across all figures, the non-target maximum amplitude ratio is lower within three specific pre-stimulus ranges: “0–200 ms”, “400–600 ms”, and “800–900 ms”. Each of these ranges aligns with the initial 0–200 ms range before any visual stimulus.

This suggests that using a stable EEG baseline voltage, such as within the 0–200 ms range, rather than relying on a single-time-point baseline, could enable more effective baseline correction. Consequently, using this stable range as a baseline would likely yield the highest accuracy in P300 peak detection.

### 3.4. Summary of Single-Time-Point Baseline Correction for P300 Peak Detection

We demonstrated that using the 0–200 ms pre-stimulus period as a relatively stable EEG baseline for baseline correction, combined with assuming the time position of the maximum P300 peak amplitude within the 300–370 ms range, enables effective height-based judgment of maximum amplitude values in averaged EEG data for target and non-target stimuli. This approach effectively enhances the accuracy of P300 detection and provides key insights for improving P300 detection accuracy.

## 4. Analysis 2: Characteristics of Conventional Time-Range-Averaged Baseline Correction for P300 Peak Detection

We analyze the characteristics of P300 peak detection based on time-range-averaged baseline correction—a conventional approach. Prior studies have predominantly used the average amplitude within the 0–100 ms pre-stimulus period, although some research has extended this range to the entire 0–1000 ms period before the stimulus. Therefore, we conducted a feature analysis of P300 peak detection by applying baseline correction using averaged amplitudes from each 1 ms time range between 0 and 1000 ms before the visual stimulus, beginning from 0 ms.

### 4.1. Feature Analysis of the Maximum Amplitude in P300 Peak Detection Based on Time-Range-Averaged Baseline Correction

**Figure 7:**
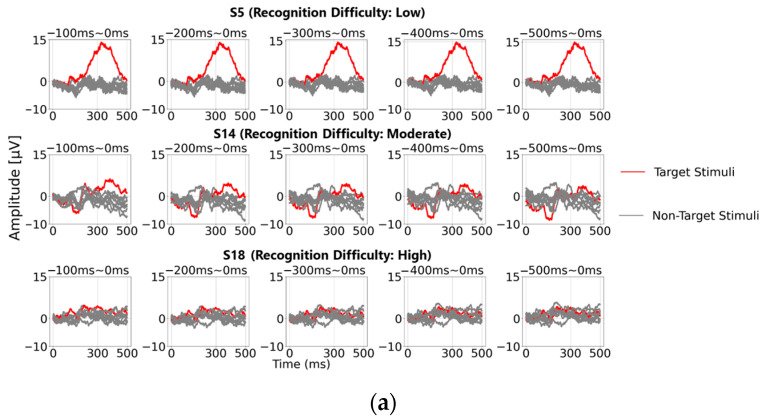

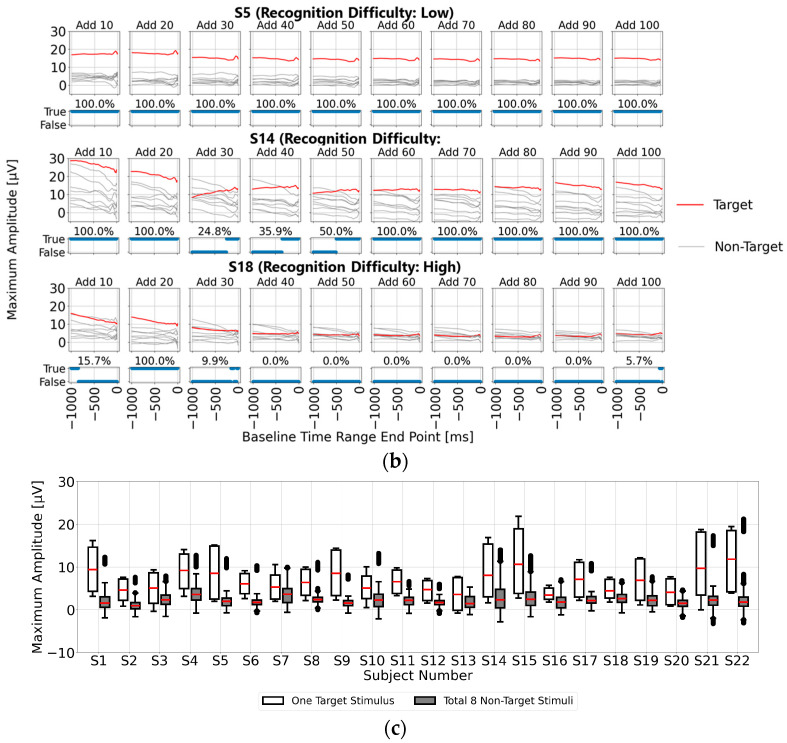
Feature analysis of P300 detection based on time-range-averaged baseline correction. (**a**) P300 peak waveforms for three participants based on five representative time-range-averaged baselines (conventional method), averaged over 100 trials; (**b**) maximum amplitudes across all baseline points for three participants using time-range-averaged baseline correction (top) and correct/incorrect classifications when the maximum amplitude for target stimuli exceeds that of non-target stimuli (bottom), visualized as blue bars labeled “True” for correct and “False” for incorrect classifications and displayed for various averaging trial counts; (**c**) box plot of maximum amplitude values for target and non-target stimuli for all 22 participants. The black markers and whiskers represent outliers and the data range excluding outliers, respectively.

Figure 7a illustrates the P300 peak detection results based on baseline correction using averaged amplitudes across five representative pre-stimulus time ranges: “0–100 ms”, “0–200 ms”, “0–300 ms”, “0–400 ms”, and “0–500 ms” (averaged over 100 trials). The top row shows data from participant S5 (“Recognition Difficulty: Low”), the middle row from S14 (“Recognition Difficulty: Moderate”), and the bottom row from S18 (“Recognition Difficulty: High”). In the case of S5, a prominent P300 peak waveform appeared, clearly distinguishing EEG data from the target and non-target stimuli. However, for S14 and S18, a wider baseline time range led to higher maximum amplitudes for the non-target stimuli than for the target stimuli, resulting in incorrect P300 detection. This discrepancy likely occurred because, as indicated in Section 3.3, the 0–200 ms range is the most stable EEG baseline voltage.

Next, following the methodology in Figure 4 from Section 3.1, we calculated the average amplitude across each 1 ms interval from 0 to 1000 ms pre-stimulus for baseline correction. We then extracted the maximum amplitude within the 200–400 ms range post-stimulus (300 ± 100 ms). These results are displayed as a graph, with the *x*-axis representing the baseline time range (showing the endpoint) and the *y*-axis representing the maximum amplitude. Below this graph, we show correct/incorrect classification results and accuracy rates for each baseline time range. The results are presented for averaging trial counts of 10, 20, 30, 40, 50, 60, 70, 80, 90, and 100. Overall, the findings indicate that, when baseline correction was performed using the 0–200 ms time range, the maximum amplitudes of the target stimuli tended to be higher than those of the non-target stimuli. However, when the baseline correction included time ranges beyond 200 ms, the maximum amplitudes of both the target and non-target stimuli increased as the baseline range widened. This trend can be attributed to baseline bias errors caused by EEG amplitude fluctuations induced by other stimuli, particularly within the 200–400 ms and 600–800 ms ranges pre-stimulus, as discussed in Section 3.3. These fluctuations are assumed to accumulate as baseline bias errors, contributing to the observed increase in maximum amplitude values.

Finally, Figure 7c shows a statistical summary (box plot) of the maximum amplitude values for the target (one stimulus) and non-target stimuli (eight stimuli) based on EEG data averaged over 100 trials for all 22 participants. Upon comparing Figure 4c and Figure 7c (from single-time-point baseline correction), it is evident that the range of the maximum amplitude values is narrower in Figure 7c. This outcome suggests that using the average amplitude over a time range for baseline correction results in smoother data compared to single-time-point baseline correction. However, because the mean only represents a central value, it does not necessarily indicate an optimal EEG baseline voltage within the 0–200 ms range before the stimulus. Conversely, these findings indicate that there is room to explore alternative methods beyond simple mean amplitude calculation to enhance the effectiveness of P300 peak detection based on insights from single-time-point baseline correction.

### 4.2. Detailed Analysis of P300 Peak Detection in the 300–370 ms Time Range for Time-Range-Averaged Baseline Correction

Based on the findings in Section 3.3, where detecting the P300 peak as the maximum amplitude within the 300–370 ms post-stimulus range was identified as the most effective approach, this section applies this time range to the time-range-averaged baseline correction for P300 peak detection.

**Figure 8:**
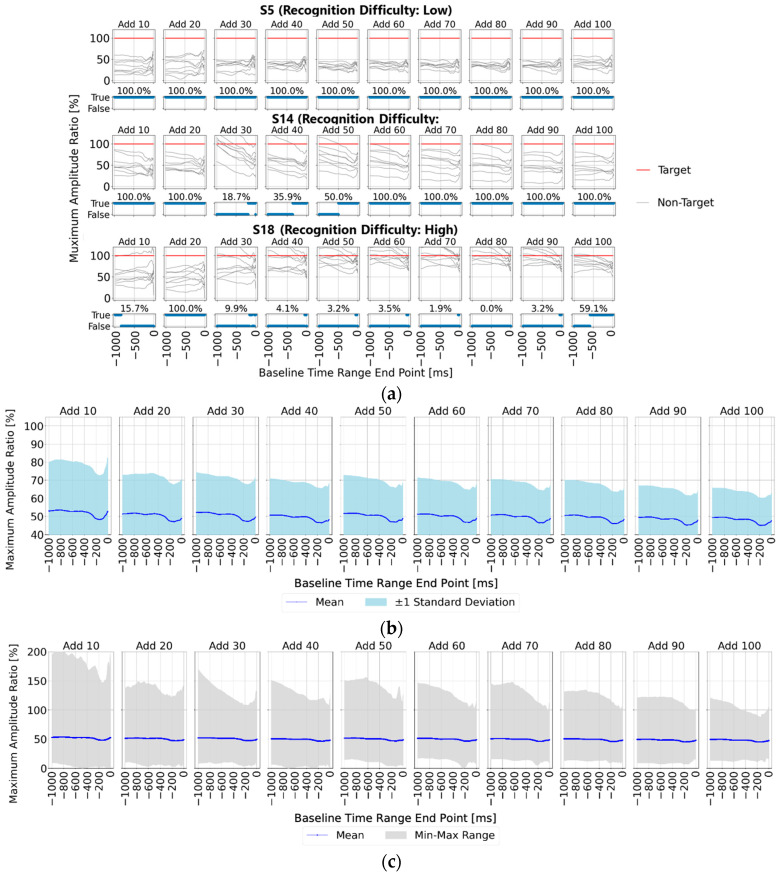
Detailed comparison of the maximum amplitudes for target and non-target stimuli with the P300 detection time range set to 300–370 ms (time-range-averaged baseline correction). (**a**) Normalized maximum amplitude values for non-target stimuli relative to the target stimulus maximum amplitude across different averaging trial counts for three participants. The correct/incorrect classifications based on these values are visualized as blue bars labeled “True” for correct and “False” for incorrect classifications; (**b**) statistical representation (mean and standard deviation) of the normalized maximum amplitude values for non-target stimuli relative to target stimuli for all 22 participants; (**c**) statistical representation (mean, maximum, and minimum) of the normalized maximum amplitude values for non-target stimuli relative to target stimuli for all 22 participants.

First, Figure 8 displays the ratio of maximum amplitude values for non-target stimuli relative to target stimuli (set to 100%) across different baseline time ranges (*x*-axis), facilitating the observation of non-target stimulus influence. The upper section of the figure shows the results for averaging trial counts of 10, 20, 30, 40, 50, 60, 70, 80, 90, and 100, while the lower section presents correct/incorrect classifications and accuracy rates for each baseline time range. Similar to Section 4.1, results are presented for participants S5, S14, and S18. The results generally follow the trends discussed in Section 3.3; however, a notable finding is that with 30 or more averaging trials, all participants exhibited lower maximum amplitudes for the non-target stimuli than for the target stimuli within the 0–200 ms pre-stimulus range, indicating a stable EEG baseline voltage.

Next, Figure 8b,c present statistical analyses across all 22 participants, showing the ratio of maximum amplitude values for non-target stimuli to those for target stimuli (set to 100%). Figure 8b displays the mean and standard deviation, while Figure 8b illustrates the mean, maximum, and minimum values within shaded areas. Across all figures, the non-target maximum amplitude ratio is lower within the pre-stimulus range of “0–250 ms”, indicating that, similar to the single-time-point baseline correction, the 0–200 ms pre-stimulus period represents a relatively stable EEG baseline voltage.

However, when using the average amplitude over a time range for baseline correction, it merely smooths the signal rather than confirming the effectiveness of the baseline correction. Therefore, it remains uncertain whether this approach consistently achieves effective baseline correction compared to single-time-point baseline correction.

### 4.3. Detailed Analysis of Baseline Range Start Time Variation

In the previous section, baseline correction was performed for all time ranges starting at 0 ms and ending at 1000 ms. As a result, the most stable time range for detecting P300 was found to be 0 ms to 200 ms. However, since a time range of 200 ms may be effective, we fixed it in this section at this value and performed baseline correction in 10 ms increments from 0 ms to −300 ms as the starting point. Then, we evaluated the results regarding P300 detection.

**Figure 9:**
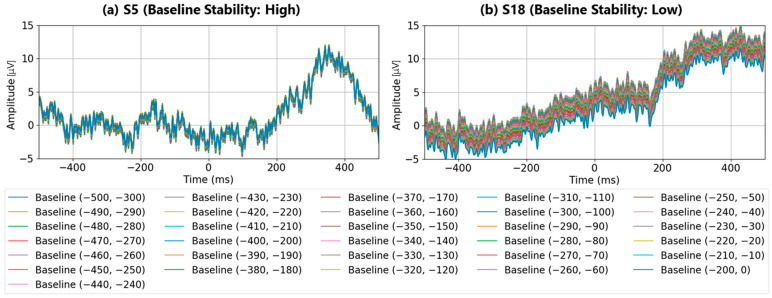

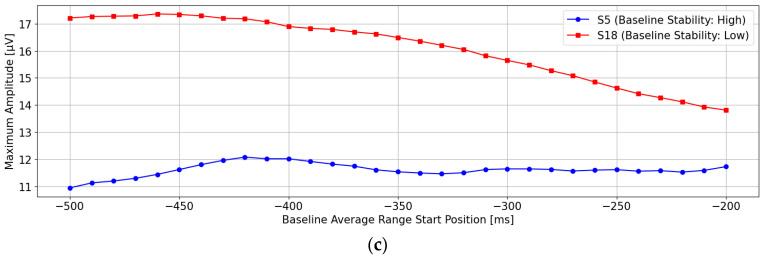
Comparison of subjects with high (S5) and low (S18) baseline stabilities in time-range-averaged baseline correction. The baseline range was fixed at 200 ms, and its starting point was shifted from 0 ms to −300 ms in 10 ms increments. (**a**) EEG baseline waveforms before and after visual stimulation for subject S5 (high detection accuracy); (**b**) EEG baseline waveforms for subject S18 (low detection accuracy); (**c**) comparison of P300 peak amplitude variations across the specified time ranges for both subjects.

Figure 9a shows the baseline EEG data before and after visual stimulation for subject S5 with a high P300 detection rate in the specified time ranges, while Figure 9b shows the baseline EEG data before and after visual stimulation for subject S18 with a low P300 detection rate in the specified time ranges. As shown in the result for S5, if the period of the waveform is within the specified time range, the baseline variation is small, even if the time range shifts. However, as shown in the result of S18, when there are waveforms with periods that exceed the specified time range, shifting the time range results in large baseline fluctuations. Comparing the maximum amplitude values of the P300 peak for each specified time range (Figure 9c), it was confirmed that subject S5, who has a high P300 detection rate, has a peak amplitude variation of approximately 1 μV, while subject S18, who has a low P300 detection rate, has a peak amplitude variation of approximately 4 μV. These results indicate that the waveforms used for P300 analysis so far should be eliminated not only for sudden fluctuations such as eye electric signals, but also for waveform characteristics with a period exceeding the specified time range of 200 ms.

### 4.4. Summary of Time-Range-Averaged Baseline Correction for P300 Peak Detection

We conducted a detailed analysis of P300 peak detection results based on time-range-averaged baseline correction. Consistent with the previous section, we confirmed that the EEG baseline potential in the 0 ms to 200 ms pre-stimulus period was relatively stable. It was also confirmed that evaluating whether the waveform period falls within this 200 ms time range is important. The reason for this is that when waveforms with periods longer than the specified time range exist, there is a drift in the 0 to −200 ms portion of the visual stimulus, making it difficult to calculate an appropriate EEG reference potential.

## 5. Analysis 3: Feature Analysis of the Proposed Multi-Time-Point Baseline Correction for P300 Peak Detection

Conventionally, P300 peak detection is performed using a single EEG baseline potential value obtained through either single-time-point baseline correction or time-range-averaged baseline correction. In this section, we validate an alternative multi-time-point baseline correction method. In this approach, multiple EEG baseline potential values are calculated within a specific time range, preliminary P300 peaks are detected for each value, and then all preliminary detection results are integrated to determine the final P300 peak.

### 5.1. Workflow of the Proposed Multi-Time-Point Baseline Correction for P300 Peak Detection

**Figure 10:**
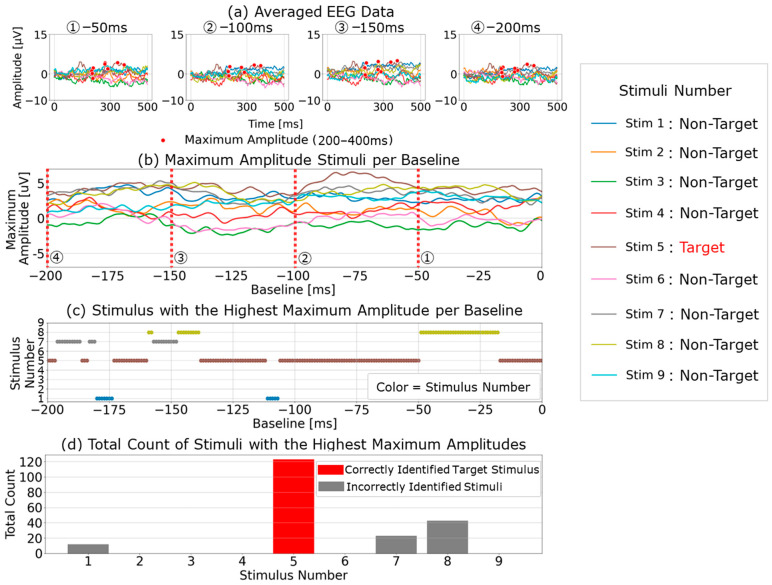
Analysis procedure for the proposed multi-time-point baseline correction method for P300 detection. (**a**) EEG waveforms for each visual stimulus after averaging at baseline points of −50, −100, −150, and −200 ms; (**b**) the maximum amplitude values for each stimulus after single-time-point baseline correction across all time points are shown. The red dashed lines (①, ②, ③, ④) in (**b**) correspond to the baseline points at −50 ms, −100 ms, −150 ms, and −200 ms, respectively, as shown in (**a**); (**c**) identification of the visual stimulus type that exhibited the highest maximum amplitude across nine types of visual stimuli, based on single-time-point baseline correction for all time points; (**d**) a cumulative count of the maximum amplitude detections for each visual stimulus across all time points, as derived from (**c**).

The workflow of the proposed multi-time-point baseline correction is shown in Figure 10. Using each 1 ms interval from 0 to 200 ms before the visual stimulus, we applied single-time-point baseline correction at each time point and calculated the maximum amplitude within the 300–370 ms post-stimulus range for the averaged EEG data (Figure 10a). We then identified the EEG data with the highest maximum amplitude among the nine different visual stimuli presented (Figure 10b) and tentatively designated it as the target stimulus (Figure 10c). Next, we aggregated the preliminary P300 peak detection results (a total of 200 points) based on single-time-point baseline correction for each interval from 0 to 200 ms pre-stimulus. The stimulus with the highest number of preliminary target identifications was ultimately classified as the final target stimulus (Figure 10d). The determined P300 peaks were classified as correct/incorrect by comparing the final identified target stimulus (based on the highest number of preliminary target identifications across 200 baseline-corrected signals) with the actual target stimulus presented during the trial. If the identified stimulus matched the actual target, it was considered a correct classification; otherwise, it was counted as incorrect. The overall classification accuracy was then calculated by dividing the number of correct classifications by the total number of trials. In summary, in this method, baseline correction and preliminary P300 detection are performed at different amplitude values within a specific time range, incorporating the EEG baseline potential width characteristics obtained from these 200 time points into the P300 detection process.

To clarify the processing steps of the proposed method, we provide the following formal description.

1. Baseline Correction:

The baseline is evaluated at multiple time points within the baseline period (e.g., –200 to 0 ms). Let $i\in \left\{1,\;2,\ldots ,N\right\}$ be the index of each visual stimulus, where *N* is the total number of stimuli (e.g., nine in this study). For each time point $t_{\mathrm{baseline}}$ in this period, the baseline-corrected EEG signal corresponding to stimulus i is computed as:

(1) $$S_{\mathrm{corrected},i}\left(t\right)=S_{i}\left(t\right)-S_{i}\left(t_{\mathrm{baseline}}\right),$$

where $t_{\mathrm{baseline}}\in [-200,0]$.

2. Frequency of Maximum Amplitude:

For each time point $t_{\mathrm{baseline}}$, the stimulus $i_{t}$ with the highest P300 amplitude within the expected time window (300–370 ms) is identified using:

(2) $$i_{t}=\arg\;\underset{i}{\max}\left(\underset{t\in \left[300,370\right]}{\max}S_{\mathrm{corrected},i}\left(t\right)\right).$$

Then, the frequency $C_{i}$ of each stimulus is selected as the maximum is determined over the entire baseline period:

(3) $$C_{i}=\sum_{t\in \left[-200,0\right]}\delta \left(i_{t},i\right)$$

where the Kronecker delta function is defined as:

(4) $$\delta \left(i_{t},i\right)=\left\{\begin{array}{l} \;\;\;\;\;1,if\;i_{t}=i,\\ \;\;\;\;\;0,\;otherwise.\; \end{array}\right.$$

3. Target Stimulus Identification:

Finally, the stimulus with the highest frequency is identified as the target:

(5) $$i^{*}=\arg\underset{i}{\max}C_{i}$$

### 5.2. Performance Comparison of P300 Peak Detection Based on Three Baseline Correction Approaches

**Figure 11:**
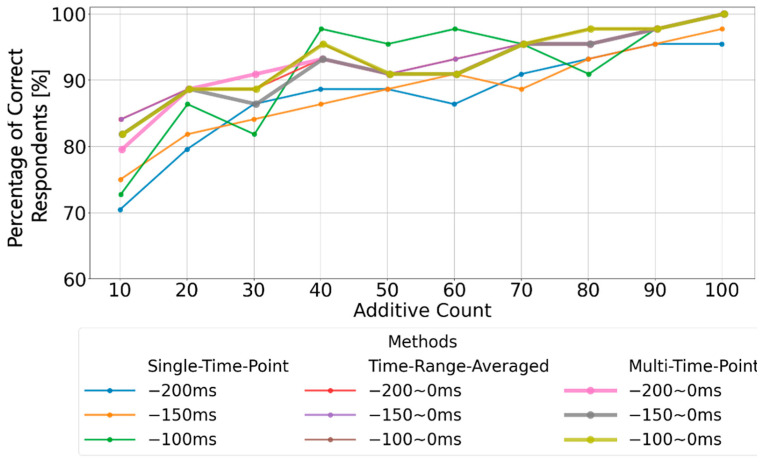
Comparison of the proportion of correct respondents (%) using single-time-point baseline correction (conventional method), time-range-averaged baseline correction (conventional method), and multi-time-point baseline correction (proposed method), using data from all 22 participants.

Finally, Figure 11 presents a comparison of the proportion of correct respondents for P300 peak detection at different averaging counts using the three baseline correction methods: single-time-point baseline correction, time-range-averaged baseline correction, and multi-time-point baseline correction. The results show that single-time-point baseline correction greatly affected the accuracy of P300 peak detection when oscillations such as basic rhythm remained in the averaged EEG data from 0 ms to 200 ms before the visual stimulus. On the other hand, time-range-averaged baseline correction and multi-time point baseline correction achieved over 90% correct responses when averaging was performed at least 40 times. In the case of the time-range-averaged baseline correction, even if the oscillations of the basic rhythm remained in the averaged EEG data from 0 ms to 200 ms before stimulation, the average amplitude value was an appropriate EEG reference potential. In multi-time-point baseline correction, provisional P300 peak detection results were calculated after the EEG reference potential was calculated from each time point, and the final result was almost the same as that obtained via time-range-averaged baseline correction, based on the overall judgment of all time point results. In addition, statistical analysis was conducted using the Wilcoxon signed-rank test to evaluate the significance of the differences in P300 detection rates obtained from the three baseline correction methods. The samples were paired because the comparisons involved the application of different analysis methods to the same EEG data; however, the normality of the differences could not be assumed. Accordingly, the Wilcoxon signed-rank test, a non-parametric method commonly used in recent P300 research [28,29,30,31], was deemed appropriate. As a result, it was found that the multi-time-point baseline correction yielded a significantly higher correct detection rate compared to the single-time-point baseline correction (W = 13.5, Holm-adjusted *p* = 0.016, effect size r = 0.56). On the other hand, no significant difference was observed between the multi-time-point baseline correction and the time-range-averaged baseline correction (W = 2.5, Holm-adjusted *p* = 0.089, r = 0.36), indicating that both methods demonstrated comparable performance.

Although the utility of both the multi-time-point and time-range-averaged baseline correction was validated, based on the results shown in Figure 11, it can be inferred that the time-range-averaged baseline correction is effective when dealing with EEG oscillations whose periods are shorter than the specified time range. However, when oscillations with periods longer than the specified range are present, it is likely that the calculated baseline value deviates from the appropriate baseline, potentially leading to errors. In contrast, the multi-time-point baseline correction, which calculates multiple baseline values within the 0 ms to 200 ms pre-stimulus window and comprehensively evaluates provisional P300 peak detections, achieved performance comparable to the time-range-averaged baseline correction under the experimental conditions. Therefore, it is suggested that further improvements in P300 peak detection accuracy may be achievable by introducing a more versatile calculation method for EEG baselines based on the oscillatory characteristics of the averaged EEG data in the 0–200 ms pre-stimulus window.

## 6. Discussion

Detailed analyses were performed using two conventional methods: single-time-point baseline correction and time-range-averaged baseline correction. The results demonstrate that, firstly, single-time-point baseline correction is not stable because differences in fundamental rhythm amplitudes arise depending on the chosen time point. Secondly, the time-range-averaged baseline correction method utilizes a single averaged amplitude within a specified time range. As a result, if waveforms with periodicities longer than the averaging window are present, it may fail to accurately estimate the waveform’s center axis, thereby resulting in an inappropriate baseline. Therefore, in this paper, we propose a multi-time-point baseline correction approach that comprehensively evaluates waveform characteristics within a specified time range. This method successfully demonstrated a performance comparable to that of the time-range-averaged baseline correction method. Additionally, since the proposed method can be regarded as a multivariate analysis of waveform characteristics within a certain time range, future enhancements to waveform evaluation methods within specific time windows have the potential to improve the performance further compared to traditional average-amplitude baseline correction.

However, a major drawback of the multi-time-point baselining approach is its higher computational cost. In this study, single-time-point correction, averaging processes, and P300 peak detection were performed at every millisecond interval from 200 ms to 0 ms before stimulus onset. It was inferred that the computational cost is greater than that of the conventional single-time-point baseline correction and time-range-averaged baseline correction methods. To further quantify these computational costs, additional experiments comparing calculation costs among the three baseline methods were conducted. Given that PC conditions can influence the computation time, each P300 analysis was performed ten times. Table 1 presents the results regarding computational costs related to the number of averaged epochs used for P300 peak detection based on each of the three baseline methods. Defining a single trial as an EEG data analysis for P300 detection following visual presentation of one target stimulus and eight non-target stimuli, the computation time per trial was approximately 0.85 ms for single-time-point baseline correction, 1.00 ms for time-range-averaged baseline correction, and 150.0 ms for multi-time-point baseline correction. Clearly, multi-time-point baseline correction entails significantly greater computational costs. Nevertheless, considering that each trial lasts 3.6 s (nine stimuli each presented sequentially for 400 ms), the 150 ms multi-time-point baseline correction process can still be executed concurrently. Thus, it is feasible to develop a real-time analysis algorithm that outputs final trial results approximately 150 ms after the last stimulus.

In conclusion, real-time P300 detection is achievable following detailed waveform analyses within specific time windows, emphasizing the importance of enhancing processing methods within these time windows beyond simplistic averaging approaches such as traditional time-range-averaged baseline correction.

This study focuses on the research of P300 interfaces for welfare applications. The primary motivation for this focus is that, for individuals with complete quadriplegia (such as patients with Locked-in Syndrome), who are unable to move any motor organs and can only recognize information through sensory inputs processed in the brain, brain-computer interfaces (BCIs) represent the only viable means of life support [32]. Among various BCIs, non-invasive BCIs that do not require surgical procedures have advanced significantly, with P300 interfaces utilizing event-related potentials (P300) measured by EEG being one of the most practically realized technologies to date [33]. The P300 Speller is a representative system based on the elicitation of P300 event-related potentials through visual stimuli. In its standard configuration, multiple characters are displayed on a screen and randomly flash one by one at 400 ms intervals. By focusing their gaze on a specific target stimulus, users can generate a P300 response that can be analyzed to identify the intended selection [17]. Previous studies have not only employed simple character stimuli but also explored the use of various visual designs to enhance practical usability [34]. Other studies have compared the detection performance of P300 potentials under variations in flash intensity, flash color, flash shape, and flash duration [35,36,37,38]. Furthermore, efforts have been made to improve the efficiency of the P300 signal analysis process itself by modifying baseline correction and signal averaging procedures traditionally based on stimulus timing. Specifically, methods that omit baseline correction and average in favor of frequency-domain analysis [25], as well as machine learning-based approaches for direct P300 peak detection [39], have been proposed.

However, in frequency analysis approaches, it becomes difficult to accurately identify P300 peaks because variations in the EEG baseline reference can cause low-frequency components to be erroneously interpreted as signal peaks. Similarly, machine learning-based methods have been reported to yield better performance when applied after baseline correction [40], underscoring the importance of identifying an appropriate “EEG baseline reference” for reliable P300 detection. Moreover, for welfare applications, EEG measurements must often be conducted in office environments rather than shielded rooms typically used in neuroscience laboratories, making them more susceptible to commercial power line noise. Therefore, establishing a baseline reference suitable for real-world measurement environments is crucial [41].

Against this background, the present study aims to clarify the relationship between continuous visual stimuli and the EEG baseline reference used in conventional P300 spellers. One distinctive feature of the P300 speller is that more than 80 flashes of visual stimuli are necessary to determine a single selection. To mitigate user fatigue and maintain concentration, the flashing interval is shortened to 200–400 ms, which is shorter than those generally used in conventional neuroscience studies. This raises concerns regarding the stability of EEG signals under such continuous stimulation conditions. Previous research has typically adopted methods where the amplitude at a single point in time (100 ms or 200 ms before stimulus onset) is used as the baseline reference, or where the average amplitude over a time window from 0 to 200 ms before stimulus onset is used. However, no studies have quantitatively validated the effectiveness of these baseline correction methods under continuous stimulation conditions. Therefore, in this study, experiments were conducted with 21 participants, using nine visual stimuli that flashed randomly at 400 ms intervals. The P300 detection performance was evaluated through comparison of the following three baseline correction methods:

1. A method based on the amplitude at each individual time point;
2. A method based on the average amplitude over specific time windows;
3. A novel method is proposed in this study, which performs a comprehensive evaluation by individually correcting the baseline across all time points from 0 to 1000 ms prior to stimulus onset.

The experimental results revealed that baseline correction based solely on a single-point amplitude is highly susceptible to fluctuations in basic EEG rhythms, leading to unstable P300 detection performance. In contrast, the method based on the average amplitude over a time window demonstrated that using the average amplitude between 0 and 200 ms before stimulus onset provided the most stable baseline reference and yielded high P300 detection performance. However, it was also observed that the stability of the baseline reference deteriorated when artifacts with periods longer than 200 ms were present.

Moreover, the proposed method involving multi-time-point baseline correction and comprehensive evaluation achieved a comparable high P300 detection performance when focusing on the 0–200 ms pre-stimulus window. From these results, it is suggested that under continuous visual stimulation with 400 ms intervals, baseline correction using the average amplitude over the 0–200 ms pre-stimulus period is most appropriate. Baseline correction based on a single-point amplitude is considered unsuitable due to the increased risk of erroneous P300 detection caused by fluctuations in basic EEG rhythms. Future work should include the prior assessment of waveform characteristics during the 0–200 ms pre-stimulus window. If only basic rhythms are present, calculating the central axis of the waveform may be appropriate. If artifacts with longer periods are detected, it may be preferable to either expand the time window or utilize the baseline reference obtained from the immediately preceding stimulus presentation. Nonetheless, because the optimal time window for evaluating a stable EEG baseline may vary depending on the visual stimulation interval, further detailed analyses of the relationship between the EEG baseline and P300 peaks under different continuous stimulation conditions are necessary.

## 7. Conclusions

In this study, we analyzed the performance of baseline correction methods for P300 analysis in order to improve the detection accuracy of EEG-based P300 event-related potentials for continuous sensory stimulations with durations of 400 ms that are used in welfare applications. The experimental method consisted of shining light, one at a time, on nine types of numbers aligned on a screen in 400 ms intervals and in a random order, and subjects were asked to focus on one type of number and view it 100 times. EEG data from 1000 ms before the visual stimulation to 500 ms after the visual stimulation were extracted and classified, and three baseline correction methods (single-point amplitude, time-range-averaged amplitude, and multiple-point amplitude correction) were applied. Characteristic evaluation was attempted based on the P300 peak detection accuracy after additive averaging. The results show that the P300 peak detection rate was highest when EEG data from 0 ms to 200 ms before visual stimulation were used for all three baseline types of stimuli. It was also found that the maximum amplitude of the target stimulus statistically occurred between 300 ms and 370 ms and that the maximum amplitude of the non-target stimulus appeared before 300 ms. In addition, we confirmed that the one-time baseline method tends to exhibit differences in detection accuracy because the amplitudes of the basic rhythm at different phase positions are selected. In addition, the maximum position of the P300 peak after additive averaging tends to fluctuate. Since time-range-averaged baseline correction was performed over a specified time range, the center voltage of the fundamental rhythm is calculated, and the maximum position of the P300 peak after additive averaging was stabilized. The latter could be used as an appropriate EEG reference potential. However, although it is effective for basic rhythms in the time range of 0 to 200 ms, it is not an appropriate EEG reference potential when oscillations with a period of longer than 200 ms are included, reducing the accuracy of P300 peak detection. Finally, we confirmed that the newly proposed multi-time-averaged baseline correction method, through which 200 provisional P300 peak estimates can be evaluated by applying single-time-point baselining to 200 time points from 0 ms to 200 ms before the visual stimulation, shows a similar performance to time-range-averaged baselining. In particular, this method can be used to evaluate the amplitude characteristics within a specified time range, with more variables than a single average value, and future improvements should lead to more accurate calculation of EEG reference potentials.

## Figures and Tables

**Table 1:** Average processing time (in milliseconds) required for each baseline correction method across different averaging counts. The values represent the mean ± standard deviation, calculated by repeating the same computation 10 times per subject and averaging across 22 participants.

Additive Count	Multi Time Point (ms)	Single Time Point (ms)	Time Range Averaged (ms)
10	1395.6 ± 25.9	8.3 ± 0.3	9.5 ± 0.3
20	2590.6 ± 33.9	14.1 ± 0.4	16.4 ± 0.5
30	3757.3 ± 65.7	19.9 ± 0.6	23.1 ± 0.7
40	4998.9 ± 79.3	25.5 ± 0.6	30.0 ± 0.7
50	6418.4 ± 101.3	31.7 ± 0.7	37.3 ± 0.6
60	7997.7 ± 143.2	43.8 ± 1.2	50.1 ± 1.3
70	9766.9 ± 151.4	52.0 ± 1.1	60.0 ± 1.5
80	11,672.6 ± 185.5	59.0 ± 1.0	67.9 ± 1.3
90	13,067.9 ± 224.8	65.2 ± 1.4	74.9 ± 1.5
100	15,137.4 ± 232.1	78.4 ± 1.7	90.0 ± 2.0

## Data Availability

The EEG data presented in this study are not publicly available due to privacy and ethical restrictions.
